# Lability of the pAA Virulence Plasmid in *Escherichia coli* O104:H4: Implications for Virulence in Humans

**DOI:** 10.1371/journal.pone.0066717

**Published:** 2013-06-21

**Authors:** Wenlan Zhang, Martina Bielaszewska, Lisa Kunsmann, Alexander Mellmann, Andreas Bauwens, Robin Köck, Annelene Kossow, Agnes Anders, Sören Gatermann, Helge Karch

**Affiliations:** 1 Institute of Hygiene and the National Consulting Laboratory for Hemolytic Uremic Syndrome, University of Münster, Münster, Germany; 2 Interdisciplinary Center of Clinical Research (IZKF), University of Münster, Münster, Germany; 3 Institute of Hygiene and Microbiology, University of Bochum, Bochum, Germany; University of Osnabrueck, Germany

## Abstract

**Background:**

*Escherichia coli* O104:H4 that caused the large German outbreak in 2011 is a highly virulent hybrid of enterohemorrhagic (EHEC) and enteroaggregative (EAEC) *E. coli*. The strain displays “stacked-brick” aggregative adherence to human intestinal epithelial cells mediated by aggregative adherence fimbriae I (AAF/I) encoded on the pAA plasmid. The AAF/I-mediated augmented intestinal adherence might facilitate systemic absorption of Shiga toxin, the major virulence factor of EHEC, presumably enhancing virulence of the outbreak strain. However, the stability of pAA in the outbreak strain is unknown. We therefore tested outbreak isolates for pAA, monitored pAA loss during infection, and determined the impact of pAA loss on adherence and clinical outcome of infection.

**Methodology/Principal Findings:**

*E. coli* O104:H4 outbreak isolates from 170 patients (128 with hemolytic uremic syndrome [HUS] and 42 with diarrhea without HUS) were tested for pAA using polymerase chain reaction and plasmid profiling. pAA-harboring bacteria in stool samples were quantified using colony blot hybridization, and adherence to HCT-8 cells was determined. Isolates from 12 (7.1%) patients lacked pAA. Analyses of sequential stool samples demonstrated that the percentages of pAA-positive populations in the initial stools were significantly higher than those in the follow-up stools collected two to eight days later in disease (*P*≤0.01). This indicates a rapid loss of pAA during infections of humans. The pAA loss was associated with loss of the aggregative adherence phenotype and significantly reduced correlation with HUS (*P*  = 0.001).

**Conclusions/Significance:**

The pAA plasmid can be lost by *E. coli* O104:H4 outbreak strain in the human gut in the course of disease. pAA loss might attenuate virulence and diminish the ability to cause HUS. The pAA instability has clinical, diagnostic, epidemiologic, and evolutionary implications.

## Introduction

*Escherichia coli* O104:H4 attracted attention as a highly virulent human pathogen in summer 2011 when it caused the largest outbreak of hemolytic uremic syndrome (HUS) ever recorded, which centered in northern Germany and resulted in 54 deaths [1]–[3]. This outbreak, and a much smaller French cluster [4] were epidemiologically associated with consumption of fenugreek sprouts [5]–[7] whose origin was traced back to imported seeds from Egypt [2], [3], [5]. Early genotypic analyses and whole genome sequencing of the outbreak strain demonstrated that this strain is a hybrid of enterohemorrhagic (EHEC) and enteroaggregative (EAEC) *E. coli* [8]–[14]. The outbreak strain not only harbors major virulence genes of both EHEC and EAEC [8]–[13], but also expresses phenotypes typical of both pathogroups including production of Shiga toxin 2a (Stx2a), the cardinal virulence factor of EHEC, and a “stacked-brick” aggregative adherence to cultured human intestinal epithelial cells [8], a defining characteristic of EAEC [15]. The gene (*stx*_2a_) encoding Stx2a in the outbreak strain is located in the genome of a prophage, which lysogenizes the *E. coli* O104:H4 genome [10], [13], [16], whereas loci encoding aggregative adherence are plasmid-borne [9]–[13].

The German and French outbreak strains contain three plasmids of 1.5 kb, ∼75 kb and ∼88 kb [9]–[14]. The smallest and the largest plasmids do not harbor genes encoding any known virulence factors. The 88 kb plasmid (pESBL), which is similar to the pEC_Bactec plasmid identified in several *E. coli* isolates of human and animal origin [11], [17], encodes the CTX-M-15 extended-spectrum beta-lactamase (ESBL), and a beta-lactamase TEM-1 [9]–[11], [13]. Notably, the pESBL plasmid was only identified in the German and French EHEC O104:H4 outbreak strains [9], [11]–[14], [18], but not in other sequenced EHEC O104:H4 strains, isolated before or after the 2011 outbreak [9], [13], [14]. This suggests that this plasmid might have been either recently acquired by the outbreak strain or lost by the other strains.

The 75 kb pAA plasmid of the outbreak strain resembles pAA of EAEC and contains EAEC-specific virulence loci [2], [3], [9]–[11], [13], including *aggR*, *aap*, *sepA*, the *aatPABCD* operon, and the *aggABCD* operon encoding the transcriptional regulator AggR, dispersin, the *Shigella* extracellular protein A (SepA), the dispersin transporter, and the aggregative adherence fimbriae I (AAF/I), respectively. The AAF/I fimbriae confer to EAEC and to the outbreak strain the aggregative adherence phenotype [8], [15]. It has been hypothesized [8] that the AAF/I augment adherence of EHEC O104:H4 to the intestinal epithelium, a process that facilitates systemic absorption of Stx2a, the major EHEC virulence factor involved in the pathogenesis of HUS [19]–[21] and the major virulence factor of the outbreak strain in animal models [22]. This highly avid adherence could explain the unprecedented virulence of this pathogen, in particular the high progression of the infection to HUS observed during the German (22%) and French (>50%) outbreaks [1], [4]. This hypothesis is supported by experiments in mice where the outbreak strain forms, plausibly via AAF/I fimbriae [3], [15] biofilms in the cecum, which enhances expression of virulence genes including *stx*_2a_ resulting in kidney damage [23]. However, the stability of pAA in EHEC O104:H4 is unknown. Therefore, we screened EHEC O104:H4 outbreak isolates for the presence of pAA, determined the frequency of loss of this element during illness, and investigated the impact of this loss on the adherence phenotype and clinical outcome of the infection.

## Materials and Methods

### Ethics Statement

This study was approved by the Ethical Committee of the Medical Faculty of the University of Muenster and of the Aerztekammer Westfalen-Lippe. The informed consent of the participants was not required because the data were analyzed anonymously. Our institutional review board waived the need for written informed consent from the participants.

### Patients and Strain Isolation and Characterization

EHEC O104:H4 strains were recovered from 170 patients (128 with HUS and 42 with diarrhea without HUS) during the 2011 German outbreak using previously described procedures [8], [24]. Briefly, stool samples were enriched for 4 h (37°C) in GN broth Hajna (Difco Laboratories, Detroit, MI, USA) and plated on sorbitol MacConkey agar (Becton Dickinson, Heidelberg, Germany), enterohemolysin agar (Sifin, Berlin, Germany), and ESBL agar (chromID ESBL; bioMérieux, Nurtingen, Germany). The overnight growth from the plates was washed into 0.9% NaCl solution and screened for the presence of the outbreak strain using a conventional multiplex PCR [8] or a real-time multiplex PCR targeting characteristic molecular features of the outbreak strain (*rfb*_O104_, *fliC*_H4_, *stx*_2a_) [24]. Colonies of typical morphology (dark green) were isolated from ESBL agar and verified as EHEC O104:H4 outbreak strain using the real-time multiplex PCR targeting *rfb*_O104_, *fliC*_H4_, and *stx*_2a_ [24], and a PCR that discriminates the outbreak strain from other *E. coli* O104 [25]. In addition, the isolates were confirmed as *E coli* (API 20 E, bioMerieux) and serotyped [26]. Multilocus sequence typing (MLST) was performed by sequencing internal fragments of seven housekeeping genes (*adk*, *fumC*, *gyrB*, *icd*, *mdh*, *purA*, and *recA*) [8], [27] and sequence types (ST) were assigned (http://mlst.ucc.ie/mlst/dbs/Ecoli). Stx production was determined using Vero cell assay [28]. Stx titers were defined as the highest dilutions of sterile culture filtrates that caused cytotoxicity in 50% of cells after 72 h of incubation.

### Case Definitions

Diarrhea was defined as three or more liquid stools per day, and as bloody if gross blood was noted. HUS was defined by hematocrit <30%, with smear evidence of intravascular hemolysis, thrombocytopenia (platelet count <150,000/mm^3^), and renal insufficiency (serum creatinine concentration greater than the upper limit of the normal range for age) [29].

### pAA Screening

Screening for pAA in *E. coli* O104:H4 isolates was performed using primers pCVD432/start and pCVD432/stop [30] which target the *aatA* region of pAA [8], [31]. *aat*-negative strains were further tested for the other pAA-encoded virulence loci (*aggR*, *aap*, *sepA*, and *aggC,* used as a marker for the *aggABCD* cluster) using published PCR assays [8], [31]–[34]. To detect pAA-harboring strains in stools, specimens were enriched in GN broth Hajna (4 h, 37°C) and the enrichment cultures were inoculated on ESBL agar plates. After overnight incubation the complete bacterial growth from ESBL agar was washed into 0.9% NaCl solution, boiled for 10 min and used as a template for PCR with primers pCVD432/start and pCVD432/stop [30].

### Plasmid Profiles and Plasmid Hybridization

Plasmids were isolated as described [35]. Briefly, bacteria grown overnight in 2 ml of Luria-Bertani (LB) broth were centrifuged, and the pellet was resuspended in 250 µl lysis buffer, heated (56°C, 15 min) and mixed with an equal volume of phenol:chloroform:isoamyl alcohol (25∶24:1) for plasmid extraction. The aqueous layer was recovered and plasmids were separated by 0.6% agarose gel electrophoresis. Gels were stained with ethidium bromide and visualized on a photoimager (Bio-Rad, Munich, Germany) using Quantity One® software (Bio-Rad). Plasmids from *E. coli* 39r861, NCTC 50192 (147, 63, 36, and 7 kb) (Health Protection Agency Culture Collections, Salisbury, UK) were used as size markers. Moreover, the separated plasmids were transferred to a nylon membrane and hybridized (DIG DNA Labeling and Detection Kit, Roche Molecular Biochemicals, Mannheim, Germany) with digoxigenin-labeled *aatA*, *aggR*, *aggC*, *aap*, and *sepA* probes generated with primers pCVD432/start and pCVD432/stop [30], MP2-aggR-f and MP2-aggR-r [32], aggC-f and aggC-r [31], aap-1 and aap-2 [33], and sepA-f and sepA-r [34], respectively, using PCR DIG Probe Synthesis kit (Roche Molecular Biochemicals).

### Colony Blot Hybridization

To determine proportions of pAA-positive colonies in initial and follow-up patients’ stools, serial ten-fold dilutions of stool enrichment cultures in GN broth Hajna were plated on ESBL agar plates and incubated overnight. The plates which contained 150–200 well separated colonies were transferred to a nylon membrane and hybridized with the pCVD432 probe as described above. The percentage of pCVD432-positive colonies (indicating the percentage of pAA-positive colonies) among the total number of colonies grown on each plates was calculated. The paired initial and follow-up samples were tested in parallel, immediately after recovery of pAA-negative isolates from the follow-up stools. Results were expressed as means ± standard deviations from three independent experiments.

### Cell Adherence Test

The ability of the strains to adhere to human intestinal epithelial cells was tested using the HCT-8 cell line (ATCC CCL-244) [8]. Briefly, semiconfluent HCT-8 monolayers were incubated with overnight cultures of the strains (∼10^7^ colony-forming units) for 3 h in the presence of 0.5% D-mannose (Carl Roth, Karlsruhe, Germany). The cells were intensively washed, fixed (70% ethanol), stained with 10% Giemsa (Merck, Darmstadt, Germany), and bacterial adherence patterns were examined by light microscopy (AxioImager A1; Zeiss, Jena, Germany) and photographed (AxioCam MRc camera) (Zeiss). The investigator was unaware of the pAA status of the isolates.

### Antimicrobial Susceptibility Testing

Susceptibility against ampicillin, cefuroxime, cefotaxime, cefpodoxime, ceftazidime, piperacillin/tazobactam, tigecycline, meropenem, gentamicin, amikacin, trimethoprim/sulfamethoxazole, ciprofloxacin, nitrofurantoin and fosfomycin was tested using the disk diffusion method according to the EUCAST breakpoints [36] and standard recommendations [37]. ESBL phenotype was demonstrated using the disk diffusion method with the Extended Spectrum Beta-Lactamase ID Discs (MAST Diagnostics, Merseyside, UK) performed according to the manufacturer’s instructions.

### Statistical Analysis

Statistical analysis was performed using the paired Student’s *t* test and Fisher’s exact test (EpiInfo version 7, CDC Atlanta). Two-tailed *P* values <0.05 were considered significant.

## Results

### A Subset of EHEC O104:H4 Outbreak Isolates Lack pAA-encoded Virulence Loci

EHEC O104:H4 isolates from 12 (7.1%) of 170 patients were negative in PCR with primers pCVD432/start and pCVD432/stop indicating that they lack *aatA* (Table 1, patients A to L, isolates 2). These 12 isolates also lacked the other pAA-encoded virulence loci (*aggR*, *aggC*, *aap*, and *sepA*) suggesting that they have lost part or all of the pAA plasmid. From each of these 12 patients, an earlier in illness EHEC O104:H4 isolate was also available (Table 1, isolates 1). Seven of these 12 initial isolates contained all the pAA-encoded virulence genes (Table 1, patients A to G, isolates 1), whereas the remaining five isolates lacked all these genes (Table 1, patients H to L, isolates 1).

**Table 1:** Characteristics of pAA-positive and pAA-negative EHEC O104:H4 isolates^a^ and clinical outcomes of infection in the respective patients.

Patient designation (Diagnosis)^b^	Isolate no.^c^(days)	Presence of pAA-encoded virulence loci (PCR/plasmid hybridization with probe)^d^							Plasmid profile (kb)^e^	*stx*_2a_	Stx2atiter^f^	AA^g^
		*aatA*	*aggR*	*aggC*		*aap*	*sepA*					
A (HUS)	1	+/+	+/+	+/+	+/+			+/+	88; 75	+	128	yes
	2 (7)	−/−	−/−	−/−	−/−			−/−	88	+	128	no
B (HUS)	1	+/+	+/+	+/+	+/+			+/+	88; 75	+	256	yes
	2 (3)	−/−	−/−	−/−	−/−			−/−	88	+	128	no
C (HUS)	1	+/+	+/+	+/+	+/+			+/+	88; 75	+	64	yes
	2 (2)	−/−	−/−	−/−	−/−			−/−	88	+	64	no
D (HUS)	1	+/+	+/+	+/+	+/+			+/+	88; 75	+	256	yes
	2 (4)	−/−	−/−	−/−	−/−			−/−	88	+	256	no
E (HUS)	1	+/+	+/+	+/+	+/+			+/+	88; 75	+	128	yes
	2 (8)	−/−	−/−	−/−	−/−			−/−	88	+	128	no
F (HUS)	1	+/+	+/+	+/+	+/+			+/+	88; 75	+	64	yes
	2 (2)	−/−	−/−	−/−	−/−			−/−	88	+	128	no
G (HUS)	1	+/+	+/+	+/+	+/+			+/+	88; 75	+	128	yes
	2 (5)	−/−	−/−	−/−	−/−			−/−	88	+	256	no
H (BD)	1	−/−	−/−	−/−	−/−			−/−	88	+	128	no
	2 (5)	−/−	−/−	−/−	−/−			−/−	88	+	128	no
I (D)	1	−/−	−/−	−/−	−/−			−/−	88	+	128	no
	2 (3)	−/−	−/−	−/−	−/−			−/−	88	+	128	no
J (BD)	1	−/−	−/−	−/−	−/−			−/−	88	+	64	no
	2 (6)	−/−	−/−	−/−	−/−			−/−	88	+	64	no
K (D)	1	−/−	−/−	−/−	−/−			−/−	88	+	256	no
	2 (4)	−/−	−/−	−/−	−/−			−/−	88	+	256	no
L (D)	1	−/−	−/−	−/−	−/−			−/−	88	+	128	no
	2 (5)	−/−	−/−	−/−	−/−			−/−	88	+	128	no

a The identity of all isolates as EHEC O104:H4 outbreak strain was confirmed using the multiplex real-time PCR targeting *rfb*_O104_, *fliC*_H4_, and *stx*_2a_ [24] and multilocus sequence typing, which demonstrated that all belong to ST678 typical for the outbreak strain [8].

b HUS, hemolytic uremic syndrome; BD, bloody diarrhea; D, diarrhea without visible blood.

c 1, initial isolate; 2, follow-up isolate; the number in parenthesis indicates the time interval between recovery of the initial and the follow-up isolate.

d +/+, PCR amplicon of corresponding size and hybridization signal on the 75-kb plasmid present; −/−, no PCR amplicon, no hybridization signal present.

e Sizes of plasmids in kilobase pairs (kb).

f Stx2a titers were defined as the highest dilutions of sterile culture filtrates that caused cytotoxicity in 50% Vero cells after 72 h.

g AA, aggregative adherence pattern (HCT-8 cells).

### Isolates Lacking pAA-associated Virulence Loci Lack pAA

**Figure 1:**
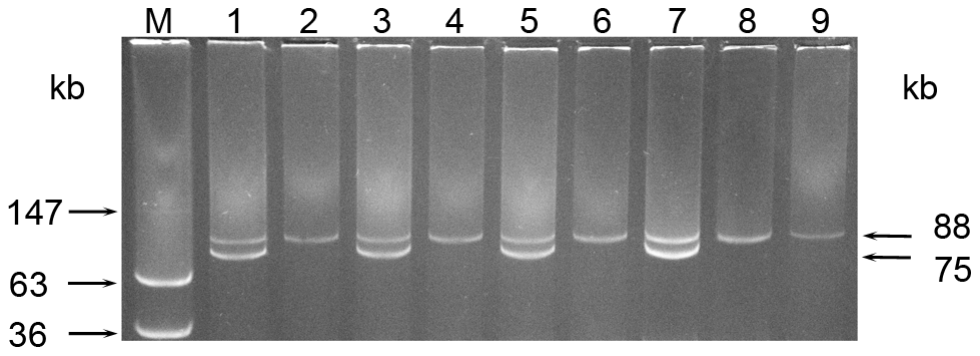
Loss of pAA by EHEC O104:H4 outbreak strain as demonstrated by plasmid profiling. Isolated plasmids were separated using 0.6% agarose gel, visualized by staining with Midori green and photographed. Lane M, molecular size marker (plasmids from *E. coli* 39r861). In lanes 1 to 10, plasmid profiles of EHEC O104:H4 outbreak isolates from the following patients are shown (patients’ designations refer to Table 1): Lane 1, initial isolate from patient A (pAA-positive); lane 2, follow-up isolate from patient A (pAA-negative); lane 3, initial isolate from patient C (pAA-positive); lane 4, follow-up isolate from patient C (pAA-negative); lane 5, initial isolate from patient G (pAA-positive); lane 6, follow-up isolate from patient G (pAA-negative); lane 7, reference EHEC O104:H4 outbreak isolate LB226692 [8]; lane 8, initial isolate from patient H (pAA-negative); lane 9, follow-up isolate from patient H (pAA-negative). Sizes of the pAA (75 kb) and pESBL (88 kb) plasmids are indicated on the right side.

To determine if the absence of the pAA-associated virulence factors resulted from the absence of pAA, we analyzed plasmid profiles of the paired initial and follow-up isolates from the 12 patients. This demonstrated that each initial isolate that contained all pAA-located virulence genes based on PCR (Table 1, patients A to G, isolates 1) possessed a 75-kb plasmid (Figure 1, lanes 1, 3, 5), which hybridized with the *aatA*, *aggR*, *aggC*, *aap*, and *sepA* probes (Table 1, patients A to G, isolates 1) confirming its pAA identity. This 75-kb plasmid was absent from all respective follow-up isolates that lacked the pAA-located virulence genes (Table 1; patients A to G, isolates 2; Figure 1, lanes 2, 4, 6). The 75-kb plasmid was also absent from both initial and follow-up isolates from patients H to L, which were all negative for pAA-encoded virulence loci by PCR (Table 1, Figure 1, lanes 8 and 9). All strains that lacked pAA harbored only a single plasmid corresponding by size (∼88 kb) to pESBL (Table 1; Figure 1, lanes 2, 4, 6, 8, 9). Accordingly, the 88-kb plasmids produced no hybridization signals with *aatA*, *aggR*, *aggC*, *aap*, and *sepA* probes (Table 1). Taken together, these results suggested that loss of pAA from the outbreak strain occurs intra-host during illness. Notably, all isolates, regardless of their pAA status, retained their *stx*_2a_ genes and abilities to produce Stx2a. Moreover, they all belonged to ST678 (Table 1) typical for the outbreak strain [8] demonstrating that the clonality was preserved in the pAA-positive and pAA-negative isolates.

### Monitoring of pAA Loss During Course of Infection

**Figure 2:**
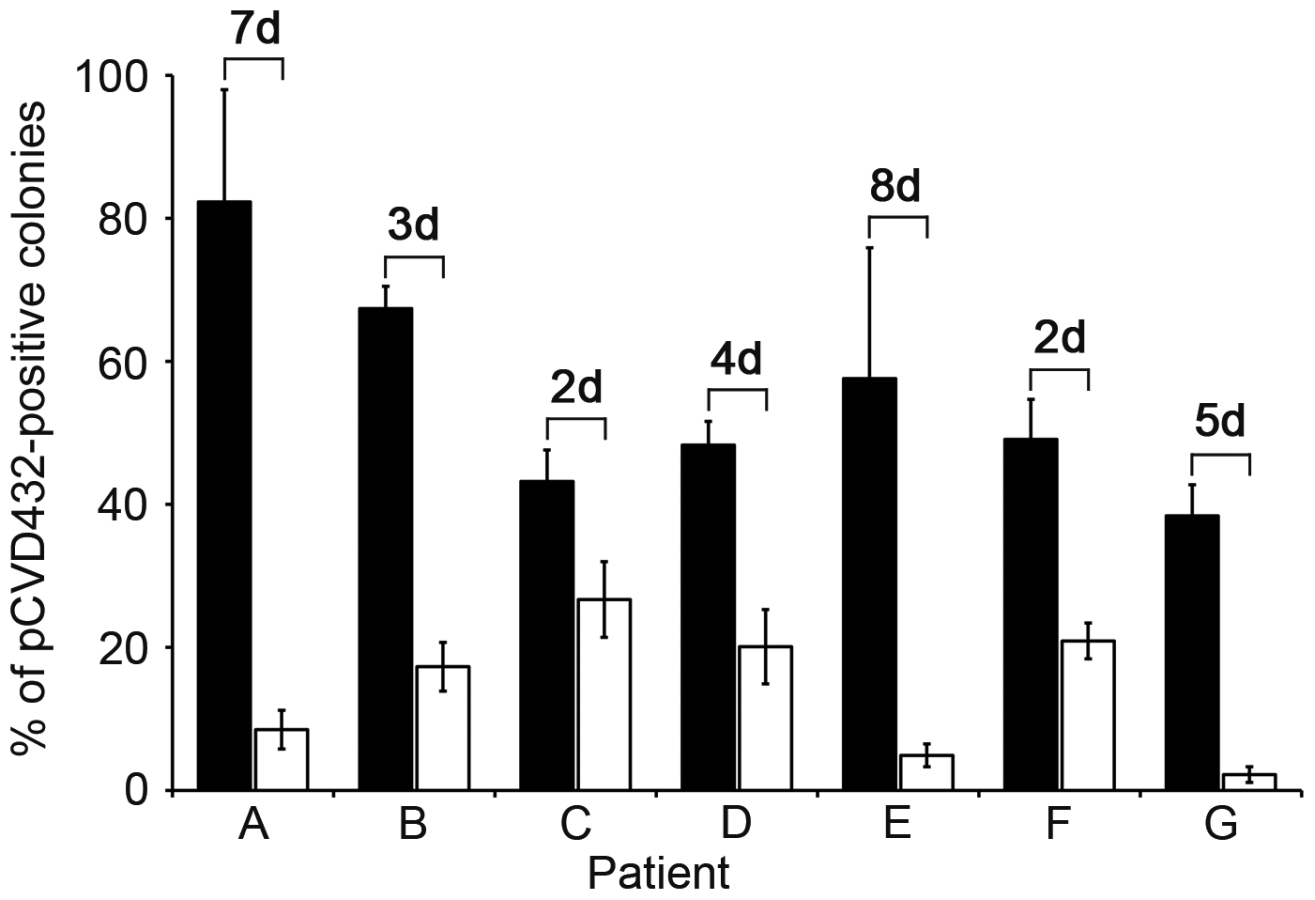
pAA is lost by the outbreak strain in the course of infection. Enrichment cultures of the initial and follow-up stool samples from patients A to G diluted to give rise to 150–200 well-separated colonies were plated on ESBL agar and pAA-positive colonies were identified using colony hybridization with the pCVD432 probe. The percentages of pCVD432-positive colonies among all colonies grown on the plates were calculated. The numbers above the columns indicate the time interval (days) between collection of the initial (black bars) and follow-up (white bars) stool samples. In all cases the percentage of pAA-positive colonies in the follow-up stool is significantly lower than that in the initial stool (*P*≤0.01; Table 2).

To more thoroughly dissect pAA loss, we first tested enriched cultures of the initial and subsequent stools from the 12 patients harvested from ESBL agar for the presence of pAA-positive bacteria using pCVD432 PCR. All samples from patients A to G, including the initial samples that yielded the pAA-positive isolates and the follow-up samples that yielded the pAA-negative isolates were positive in the pCVD432 PCR (Table 2) indicating that they all contained at least some pAA-positive bacteria. In contrast, both initial and follow-up stool samples from patients H to L, which all yielded pAA-negative isolates, were negative in the pCVD432 PCR (Table 2) indicating that pAA-positive outbreak strain was indeed absent from these stool samples. To confirm the results of this PCR pAA stool screening and to clarify the apparent discrepancy between recovery of pAA-negative isolates from the follow-up stools of patients A to G, which were PCR positive for pAA-harboring strains, we performed colony blot hybridization of the initial and follow-up stool cultures from patients A to L with the pCVD432 probe and determined the proportions of pCVD432-positive colonies. This demonstrated that in patients A to G the percentages of pCVD432-positive colonies in the follow-up stools were significantly lower than those in the initial stools (*P*≤0.01), indicating a rapid loss of pAA in the course of disease (Table 2, Figure 2). The low proportions of pAA-positive organisms within the prevailing pAA-negative outbreak strain populations in the follow-up stools (Table 2, Figure 2) are likely to be the reason for isolation of pAA-negative colonies from ESBL agar cultures of these stool samples, which was performed by random colony picking (no difference in morphologies of pAA-positive and pAA-negative colonies was observed on ESBL agar). In accordance with negative results of pCDV432 PCR screening, no pCVD432-positive colonies were found in the initial and follow-up stool samples from patients H to L (Table 2). This confirmed that stools of these patients did not contain pAA-positive organisms.

**Table 2:** Qualitative and quantitative analyses of pAA-positive bacteria in the paired initial and follow-up stool samples.

Patient designation (Diagnosis)^a^	Stool sample^b^ (days)	pAA plasmidin isolate^c^	Presence ofpAA^+^ bacteriain stool sample^d^	% of pAA^+^ colonies^e^	*P* f
A (HUS)	1	+	+	82.3	
	2 (7)	–	+	8.5	0.01
B (HUS)	1	+	+	67.4	
	2 (3)	–	+	17.3	<0.001
C (HUS)	1	+	+	43.2	
	2 (2)	–	+	26.7	0.002
D (HUS)	1	+	+	48.3	
	2 (4)	–	+	20.1	0.003
E (HUS)	1	+	+	57.6	
	2 (8)	–	+	4.9	0.031
F (HUS)	1	+	+	49.1	
	2 (2)	–	+	20.9	0.004
G (HUS)	1	+	+	38.4	
	2 (5)	–	+	2.2	0.002
H (BD)	1	–	–	0	n.a.
	2 (5)	–	–	0	n.a.
I (D)	1	–	–	0	n.a.
	2 (3)	–	–	0	n.a.
J (BD)	1	–	–	0	n.a.
	2 (6)	–	–	0	n.a.
K (D)	1	–	–	0	n.a.
	2 (4)	–	–	0	n.a.
L (D)	1	–	–	0	n.a.
	2 (5)	–	–	0	n.a.

a HUS, hemolytic uremic syndrome; BD, bloody diarrhea; D, diarrhea without visible blood.

b 1, initial stool sample; 2, follow-up stool sample; the number in parenthesis indicates the time interval between collection of the initial and the follow-up stool sample.

c +, the isolate contained all pAA-encoded virulence genes (*aatA*, *aggR*, *aggC*, *aap*, *sepA*) in PCR and harbored a 75-kb plasmid hybridizing with pCVD432, *aggR*, *aggC*, *aap*, and *sepA* probes. -, the isolate lacked all pAA-encoded virulence genes in PCR and lacked the 75-kb plasmid in plasmid profiling.

d +, an amplicon of corresponding size was obtained from the whole stool culture harvested from ESBL agar in PCR with primers pCVD432/start and pCVD432/stop [30];

-, no PCR amplicon from the whole stool culture was obtained with these primers.

e Determined by colony blot hybridization of stool cultures plated on ESBL agar with the pCVD432 probe and calculated from the total numbers of colonies grown on the plates.

f Paired Student's *t* test (*P*<0.05 considered significant); n.a., not applicable.

### Loss of pAA Leads to the Loss of Aggregative Adherence

**Figure 3:**
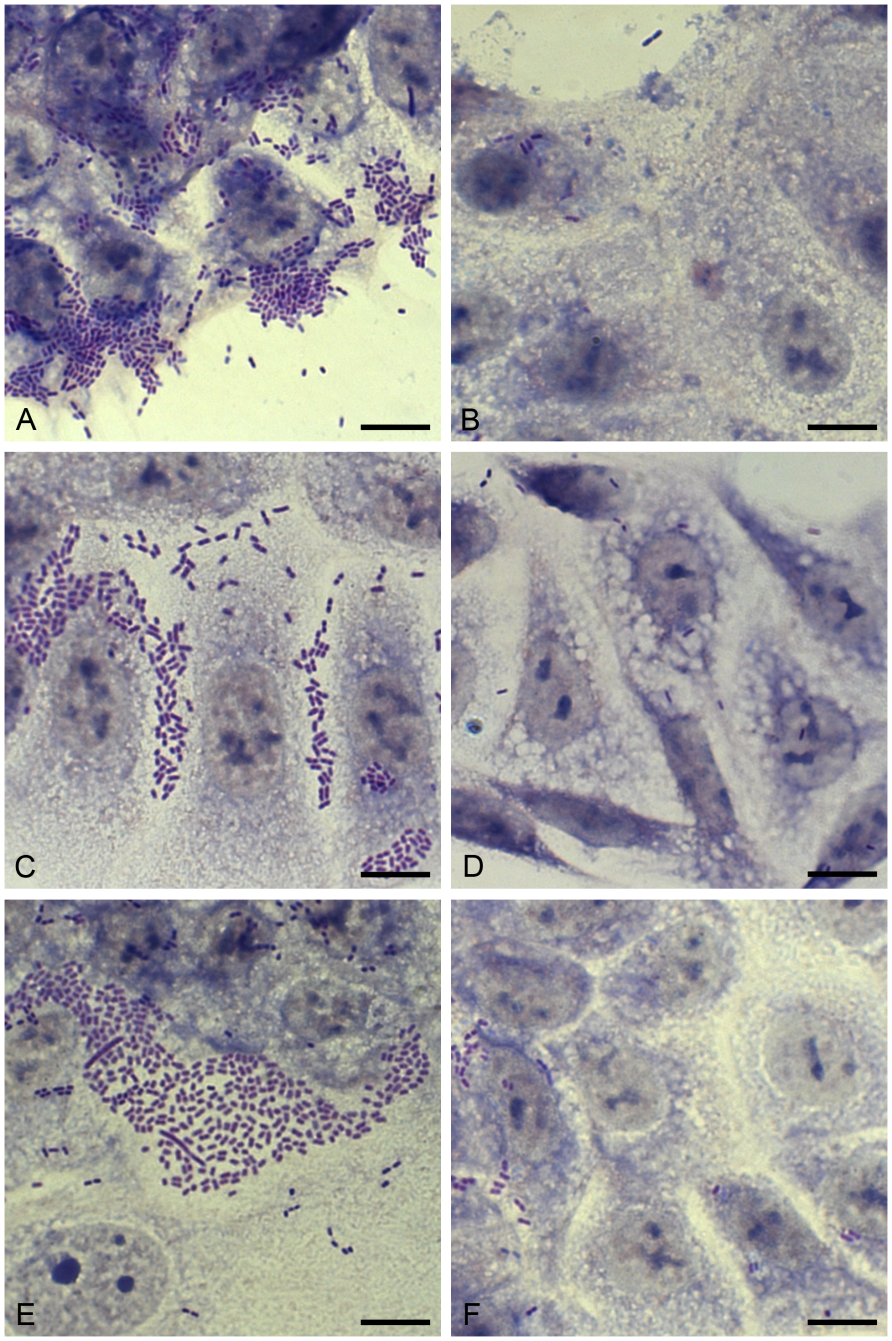
Loss of pAA by EHEC O104:H4 outbreak strain leads to the loss of the aggregative “stacked-brick” adherence to HCT-8 cells. HCT-8 monolayers were incubated with overnight cultures of initial or follow-up isolates from patients A, C and F for 3 h in the presence of 0.5% D-mannose. The cells were washed, fixed and stained with 10% Giemsa. Bacterial adherence patterns were examined using light microscopy (AxioImagerA1; Zeiss, Jena, Germany). (A, C, E) adherence phenotypes of the initial pAA-positive isolates from patients A, C, and G, respectively. (B, D, F) adherence phenotypes of the pAA-negative follow-up isolates from the respective patients are shown. Data from one of three experiments are shown. Bar = 10 µm.

Adherence phenotypes correlated perfectly with pAA genotypes. Each of the seven pAA-positive initial isolates from patients A to G produced large “stacked-brick” aggregates on HCT-8 cells (Table 1; Figure 3A, 3C, 3E), whereas the pAA-negative derivatives isolated from follow-up stools of these patients adhered weakly to these cells, usually as sparse single bacilli or small clusters consisting of three to ten bacteria (Table 1; Figure 3B, 3D, 3F). Thus, the loss of pAA by EHEC O104:H4 outbreak strain ablated the aggregative adherence of this pathogen to human intestinal epithelial cells.

### Loss of pAA Reduces the Ability of the Outbreak Strain to Cause HUS

Because the ability to intensively adhere to the intestinal epithelium might contribute to the high virulence of EHEC O104:H4 [3], [8], we asked if pAA loss might be associated with reduced virulence of this pathogen. To this end, we compared clinical outcomes of the infection in patients A to G, who had pAA-positive strains in their initial stools indicating that they were infected with a pAA-positive strain, with those in patients H to L, who had only pAA-negative organisms in their initial stools indicating that they were infected with a pAA-negative organism (Table 2). All seven patients who were infected with a pAA-positive EHEC O104:H4 (patients A to G) developed HUS, whereas all five patients who were infected with a pAA-negative strain (patients H to L) developed diarrhea without HUS, which was bloody in two cases and non-bloody in three cases (Table 2) (*P* = 0.001). Hence, as demonstrated by analyses of the available stool samples, the loss of the pAA plasmid might mitigate virulence of the EHEC O104:H4 outbreak strain, and in particular reduce its ability to cause HUS.

### Antimicrobial Susceptibility of pAA-positive and pAA-negative Isolates

All strains including the pAA-positive and pAA-negative isolates produced ESBL with resistance to all penicillins and cephalosporins tested and susceptibility to a carbapenem (meropenem). Intermediate resistance to piperacillin-tazobactam was observed in half of the strains and limited to pAA-negative isolates. All strains were susceptible to fluoroquinolones and aminoglycosides. Only one strain was susceptible to trimethoprim-sulfamethoxazole. The antimicrobial susceptibilities were identical in initial and follow-up isolates except for one patient (patient B), where the follow-up isolate was, in contrast to the initial one, resistant to trimethoprim/sulfamethoxazole and changed susceptibility to piperacillin-tazobactam to intermediate resistance (Table S1).

## Discussion

pAA, which introduces a broad repertoire of EAEC virulence genes into the EHEC O104:H4 outbreak strain [2], [9]–[12], is a relatively unstable genetic element, as demonstrated in this study by its intra-host loss during course of disease. This process has several practical implications. From a clinical standpoint, the loss of pAA plasmid in patients that had already developed HUS does not reverse the clinical outcome because the primary lesion, i.e. microvascular endothelial injury resulting from systemic absorption of Stx, had been already set. No clinical data are presently available to assess whether or not the pAA loss during the course of HUS might mitigate the severity of the disease. On the other hand, the pAA loss might diminish the ability of this pathogen to cause HUS in individuals infected with such pAA-negative strains. As indicated by data obtained by analyses of stool samples received in our laboratory, an infection with a pAA-negative derivative of EHEC O104:H4 is less likely to result in HUS. However, it should be noted that because the pAA-encoded proteins are primarily virulence factors of EAEC, which do not cause HUS [15], the absence of pAA *per se* cannot be directly linked to the reduced ability of pAA-negative variants of the outbreak strain to cause HUS. Rather, the reason for this reduced virulence of pAA-negative derivatives is probably loss of their ability to efficiently colonize the human gut, as indicated by their diminished adherence to intestinal epithelial cells in vitro (Figure 3). The substantially reduced intestinal adherence/colonization plausibly results in lack of systemic absorption of Stx2a, the major virulence factor of the outbreak strain [22] and thus lack of Stx-mediated microvascular endothelial injury, which forms the histopathological basis of HUS [19]–[21], [38]. However, without more extensive analysis of host factors that might have influenced HUS development, we are cautious in assigning pAA as a risk factor for this complication.

The relative instability of pAA in the human gut contrasts with the apparent stability of this element in vitro, as supported by only a single report of pAA loss by the outbreak strain during laboratory subcultures [14]. In addition, pAA was retained by the outbreak strain after the strain had been entered to the viable but non-culturable state using stress conditions and subsequently resuscitated [39]. Together, these observations suggest that pAA stability can differ under different environmental conditions. Therefore, because no specific therapy is presently available for EHEC infections, induction of pAA loss at a very early stage of human infection, i.e., before intestinal colonization and subsequent systemic translocation of Stx occurs, might be an approach to mitigate the clinical course of the EHEC O104:H4-mediated disease.

Although we cannot exclude the possibility that the pAA loss we observed in a subset of outbreak isolates occurred in vitro, i.e., during stool processing and culture, at least two observations argue against this scenario. The first is the stability of pAA in vitro under extreme stress conditions [39], and the second is the correlation between proportions of pAA-negative colonies in follow-up stools and the day of illness (Figure 2), indicating that the pAA loss is a time-dependent in-host process.

In contrast to the lability of pAA, the *stx*_2a_ gene of the outbreak strain, which is encoded on an inducible bacteriophage [16], [40], was stable during human infection as demonstrated by its presence in all isolates in this study. This apparent stability contrasts with that of *stx*_2a_-containing phages in other EHEC serotypes, in particular O157:H- (sorbitol-fermenting strains) and O26:H11 [41]–[45]. We cannot draw any conclusions from our data about the stability of pESBL plasmid in the EHEC O104:H4 outbreak strain because all isolates tested in this study were recovered from ESBL agar, which selects for pESBL-positive organisms.

From a diagnostic standpoint, the instability of pAA diminishes the sensitivity of molecular methods targeting pAA loci [46] to detect the outbreak strain, and for this reason, we prefer to target more stably integrated chromosomal encoded loci [24], [25], [47]. The possible absence of pAA should be considered in epidemiological studies where isolates from different origins (for example a putative source of infection and patients) are compared. Stability of pAA in foods is unknown but pAA loss has not been reported in isolates recovered from food samples [3]. Also, the rapid and significant decrease in numbers of pAA-positive EHEC O104:H4 organisms in patients’ stools during the course of infection suggests that the risk of an index case passing on severe disease to another person diminished over the course of an outbreak.

Finally, pAA loss clearly illustrates “real-time” evolution of the EHEC O104:H4 outbreak strain. The lability of pAA (and of its virulence loci) might reflect the rarity with which this particular pathogen has been identified in human disease before 2011 [2], [3], [8], [48], [49]. The influence of a loss of an important virulence locus on the pathogen’s virulence as demonstrated in our study for the EHEC O104:H4 2011 outbreak strain serves as a paradigm for a possible regulation of population-based virulence in other emerging pathogens.

## Supporting Information

Table S1 Antimicrobial susceptibilities of pAA-positive and pAA-negative EHEC O104:H4 isolates.
(DOC)
