# Supplementary material for: Lability of the pAA Virulence Plasmid in Escherichia coli O104:H4: Implications for Virulence in Humans
Source: PLoS One. 2013 Jun 21;8(6):e66717. doi: 10.1371/journal.pone.0066717 (PMC3689698; doi:10.1371/journal.pone.0066717)
Supplement: Table S1 — Antimicrobial susceptibilities of pAA-positive and pAA-negative EHEC O104:H4 isolates. (DOC) [file pone.0066717.s001.doc]

**Table S1.** Antimicrobial susceptibilities of pAA-positive and pAA-negative EHEC O104:H4 isolates

| Pat-ient | Iso-late no. | pAA | Susceptibility to antimicrobial^a^ | | | | | | | | | | | | | | |
| --- | --- | --- | --- | --- | --- | --- | --- | --- | --- | --- | --- | --- | --- | --- | --- | --- | --- |
|  |  |  | AMP | CXM | CTX | CPD | CAZ | MEM | TZP | TGC | CN | AK | STX | CIP | F | FOT | ESBL^b^ |
| B | 1 | + | R | R | R | R | R | S | S | S | S | S | S | S | S | S | + |
|  | 2 | - | R | R | R | R | R | S | I | S | S | S | R | S | S | S | + |
| E | 1 | + | R | R | R | R | R | S | S | S | S | S | R | S | S | S | + |
|  | 2 | - | R | R | R | R | R | S | S | S | S | S | R | S | S | S | + |
| G | 1 | + | R | R | R | R | R | S | S | S | S | S | R | S | S | S | + |
|  | 2 | - | R | R | R | R | R | S | S | S | S | S | R | S | S | S | + |
| H | 1 | - | R | R | R | R | R | S | I | S | S | S | R | S | S | S | + |
|  | 2 | - | R | R | R | R | R | S | I | S | S | S | R | S | S | S | + |
| L | 1 | - | R | R | R | R | R | S | I | S | S | S | R | S | S | S | + |
|  | 2 | - | R | R | R | R | R | S | I | S | S | S | R | S | S | S | + |

^a^ AMP, Ampicillin; CXM, Cefuroxime; CTX, Cefotaxime; CPD, Cefpodoxime; CAZ, Ceftazidime; MEM, Meropenem; TZP, Piperacillin/Tazobactam; TGC, Tigecycline; CN, Gentamicin; AK, Amikacin; STX, Trimethoprim/Sulfamethoxazole; CIP, Ciprofloxacin; F, Nitrofurantoin; FOT, Fosfomycin. S = susceptible, I = intermediate, R = resistant (according to the EUCAST Breakpoints [32].

^b^ Antibiotics included in the ESBL Detection Disc set (MAST): Ceftazidime 30μg; Ceftazidime 30μg + Clavulanic acid 10μg; Cefotaxime 30μg; Cefotaxime 30μg + Clavulanic acid 10μg; Cefpodoxime 10μg; Cefpodoxime 10μg + Clavulanic acid 1μg.
